# Laboratory Study on the Stiffening Phenomena Caused by Aging and by the Addition of Kraft Lignin at Low Dosages Measured by Physico-Chemical and Rheological Tests on a Soft Bitumen

**DOI:** 10.3390/ma18102209

**Published:** 2025-05-10

**Authors:** Gabriel Orozco, Sébastien Lamothe, Wesam Al-Falahat, Jean-Claude Carret, Alan Carter

**Affiliations:** Laboratoire sur les Chaussées et Matériaux Bitumineux (LCMB), Département de Génie de la Construction, École de Technologie Supérieure (ÉTS), Montréal, QC H3C 1K3, Canada; gothor@netc.fr (G.O.); sebastien.lamothe.1@ens.etsmtl.ca (S.L.); jean-claude.carret@etsmtl.ca (J.-C.C.)

**Keywords:** kraft lignin, bitumen, oxidation, rheology, infrared spectroscopy, viscoelasticity

## Abstract

This study investigates the stiffening phenomena caused by aging and low-dosage Kraft lignin addition on a soft bitumen (PG58S–28)- used in cold climate regions. Through a combination of physico-chemical and rheological analyses, including Fourier-transform infrared spectroscopy (FTIR), Brookfield rheometer viscosity (BRV), dynamic shear rheometer (DSR), multiple stress creep recovery (MSCR), bending beam rheometer (BBR), and complex shear modulus (G*) tests, the impacts of lignin modification and thermo-oxidative aging are evaluated. In particular, the anti-aging potential of lignin is scrutinized. The results indicate that while the carbonyl index effectively tracks bitumen aging, the sulphoxide index is less reliable due to high initial S=O bond content in Kraft lignin and greater repeatability variability. Standard rheological tests (BRV, DSR, MSCR, and BBR) show that long-term aging significantly increases bitumen stiffness, while lignin modification leads to a moderate stiffening effect but does not exhibit any noticeable anti-aging properties. The G* analysis confirms that aging strongly influences bitumen rigidity, particularly at low and intermediate equivalent frequencies, while lignin acts similarly to an inert filler, with minimal effects on linear viscoelastic (LVE) behaviour. Overall, the study concludes that the addition of Kraft lignin at low dosage does not alter the fundamental aging mechanisms of bitumen, nor does it provide significant antioxidant benefits. These findings contribute to the ongoing discussion on bio-based bitumen modifiers and their role in sustainable pavement materials.

## 1. Introduction

Flexible pavements make up the majority of paved roads around the world. These pavements are made from bituminous materials, the production of which requires an enormous amount of energy and generates large quantities of greenhouse gas (GHG) emissions [1]. While the use of recycled asphalt is now commonplace, and efforts are being made to reduce energy consumption by lowering the temperature of hot-mix asphalt (HMA), more is expected of our paving industry when it comes to the environment. This is why researchers are striving to replace bitumen, either totally (i.e., with a direct alternative) or partially (i.e., with a bitumen extender or modifier), with more environmentally friendly, sustainable products [2,3].

Bio-based products from organic matter are one possible tactic for replacing all or part of the bitumen [3]. Bio-based products are extracted from the biomass of bacterial, fungal, plant, or animal origin and are renewable. They can come from municipal or urban waste or from agricultural and forestry byproducts or residues.

One such bio-based product is lignin, the second most abundant biopolymer in nature [4]. Numerous lignin sources are available from plants and woods, such as hemp, jute, wood pulp, and cotton. For example, wood mainly consists of cellulose, hemicellulose and lignin [5]. The lignin content of hardwoods and softwoods varies between 18–25% and 25–35%, respectively [6,7].

In the pulp and paper industry, lignin is extracted in order to obtain stronger paper that is less sensitive to light and heat, which causes its degradation and discoloration [8], although this assertion is questioned by Małachowska et al. [9].

Lignin is insoluble in water and alcohol but soluble in weak alkaline solutions [10]. There are a multitude of lignin extraction processes (with or without pre-treatments and treatments with carbon dioxide, sulphur dioxide, sulphuric acid, alkaline, or other products) to produce the following lignin types: low-purity, lignosulfonate, Kraft, soda, organosolv, high-grade, sulphur-free, hydrolysis, and alkali lignins [7,11]. In particular, in the oldest process, known as sulphite pulping, the lignin is sulfonated to solubilize and separate it. The sulfiting process produces lignosulfonates as byproducts. In the more recent and widespread Kraft process, lignin is separated by acid precipitation. The Kraft process produces lignin with a higher phenolic compound content than initial lignin [12].

The complex chemical structure and the physical properties of lignin depend on the original source and the extraction process used [13]. Lignin is an amorphous biopolymer that appears as a sticky brown powder material with low density. Lignin is a three-dimensional, highly cross-linked macromolecule linked by carbon–carbon bonds and composed of three kinds of substituted phenols: sinapyl (C_11_H_14_O_4_), coniferyl (C_10_H_12_O_3_), and *p*-coumaryl (C_9_H_10_O_2_) alcohols [14]. It should be noted that the chemical structures of lignin and bitumen are similar since both are primarily carbon-based hydrocarbon materials [14,15]. The unique chemical and physical characteristics of lignin make it a high-potential bitumen modifier [16].

The pulp and paper industry, one of the main producers of lignin, generates 50–70 million tons of lignin per year, and by 2030, it is estimated that lignin production could increase by 225 million tons per year [17,18]. Of this large quantity, the majority of lignin is used as a fuel to generate steam or electricity. Notably, only 2% is used as bioproducts for which numerous applications have been proposed for lignosulfonates: dispersants, humectants, emulsion stabilizers, and sequestrants [12,17,19]. Kraft lignin, one of the most common types of lignin, has been used as a bitumen modifier since 1979 [20]. However, the presence of sulphur can hinder and limit the use of Kraft lignin beyond energy production. This is why the industry is looking at alternative extraction methods to make lignin sulphur-free and thus reduce the volatile organic compounds (VOCs) and odour-causing components from sulphur compounds [21,22,23].

Yet, generally, Kraft lignin addition improves the performance of bitumen at very high, high, and intermediate temperatures. This improvement is reflected in increased stiffness, as indicated by a higher viscosity (*η*) and complex shear modulus (|G*|), as well as greater elasticity, shown by a lower phase angle (*φ*). Additionally, it improves the resistance to permanent deformation under repeated stress, evidenced by a lower *J*_*nr*,3.2_ value. These effects are observed through Brookfield rotational viscometer (BRV), dynamic shear rheometer (DSR), and multiple stress creep recovery (MSCR) tests. However, at low temperatures, Kraft lignin negatively impacts performance by increasing creep stiffness (*S*(60)) and reducing creep relaxation (*m*(60)), as seen in bending beam rheometer (BBR) results [1,17,24,25,26,27,28,29,30,31,32].

BRV, DSR, MSCR, and BBR are simple rheological tests, listed in the AASHTO (American Association of State Highways and Transportation Officials) M320 standard, carried out on the bitumen at various temperatures. The performance is used to determine the mixing and compaction temperatures (*T_mixing_* and *T_compaction_*) of the mixture and the high and low temperatures (*T_high_* and *T_low_*) used to classify the bitumen according to the SUPERPAVE performance grades (PG) method.

The addition of lignin increases the viscosity at very high temperatures of the bitumen, which leads to higher *T_mixing_* and *T_compaction_*. Similarly, the lignin modification stiffens the bitumen at low, intermediate, and high temperatures, which translates into higher *T_high_* and *T_low_*_._ All these effects are significant if the lignin content exceeds more than 5 or 10% [24].

To counteract the increase in bitumen stiffness caused by lignin modification at intermediate to high dosages, a softer bitumen can be used. This is the same technological approach used to deal with the stiffening effect of reclaimed asphalt pavement (RAP), which contains hard, oxidized (aged) bitumen. This approach helps maintain good performance at low temperatures, preventing excessive brittleness in hot mix asphalt (HMA). This was carried out successfully with up to 30% lignin in a study by Al-Falahat et al. [24].

Aged bitumen in pavements (or in RAP) has gone through two aging phases: (1) short-term aging (STA), occurring at a high temperature, i.e., during the mixing, placing, and compaction of the asphalt; and (2) long-term aging (LTA), occurring at ambient temperature, i.e., during several years of service life (or the life of the pavement or the asphalt layer).

In the laboratory, STA is simulated using a rolling thin film oven (RTFO) for 85 min at 163 °C. Then, for LTA, the bitumen is aged with a Pressure Aging Vessel (PAV) for 20 h at 90, 100, or 110 °C depending on the bitumen (grade) that is suitable for the climate (cold, moderate, or hot). These standardized aging procedures are limited to thermo-oxidative aging, but other forms of aging also occur [33], such as photo-oxidation and photochemical reactions from direct or reflected exposure to light and ultraviolet radiation, aging induced by water and moisture, chemical interactions with atmospheric gases beyond oxygen, and microbiological degradation.

Simple and standard rheological tests are performed at different aging stages to evaluate bitumen properties. Specifically, the DSR is used on unaged bitumen, the MSCR test is conducted after STA, and the BBR test is carried out after LTA for bitumen grading and acceptance, following the 4101 Quebec standard (4101 Quebec standard [34], adapted from AASHTO M320). Additionally, these tests can be used at each aging stage (unaged, STA, and LTA) to compare different bitumens, with or without additives, and to quantify their aging progression over time. Based on the results of standard rheological tests, it has been shown that some lignin sources, with and without special treatments, reduce or may reduce the aging of bitumens [1,17,25,26,27,29,30,31]. However, other studies with the same rheological approach mention that the addition of lignin has no impact on aging [35].

In the province of Quebec, for soft bitumens, such as PG 52n–40 and PG 58n–34, PAV aging is extended up to 40 h in accordance with the AASHTO R28 standard and as required by the 4101 Quebec standard [34]. In particular, the difference (or delta) between the critical low temperatures (Δ*T_c,low_*) determined with the parameters *S*(60) and *m*(60) for the BBR test must be less than 5.0 °C, otherwise, the bitumens risk generating greater block cracking of bituminous pavement [36]. The Δ*T_c,low_* parameter has been proposed as a relatively simple method for measuring the loss of relaxation properties of bituminous binders [37].

Bitumen aging has multiple origins and is influenced by 15 factors, with the most significant being oxidation, the volatilization of light fractions, physical (or steric) hardening, and the loss of components through absorption by aggregates (or exudation) [33]. Oxidation intensifies as the bitumen film thickness decreases and as temperature, exposure time, and oxygen availability increase, which explains why surface layers in pavement age faster than deeper layers. Volatilization is primarily affected by film thickness, temperature, and exposure time. Since aging results from a combination of these elements, it accelerates significantly under high temperatures and prolonged exposure.

Also, following the STA with the RTFO, the mass of the bitumen must not vary by more than 1% from the initial mass (4101 Quebec standard). This variation, generated by the RTFO test (or device), makes it possible to quantify the extent of the oxidation phenomenon (increase in mass), generally that of the volatilization of the light portion (loss of mass) of the bitumen. However, this total variation does not make it possible to properly quantify the magnitude of each of these competing factors (oxidation vs. volatilization).

Oxidation is considered to be the main cause of bitumen hardening [33]. Bitumen oxidizes slowly when it comes into contact with air. In particular, oxygen from the air binds to bitumen molecules, converting low-molecular-weight molecules (maltenes: composed of saturates, aromatics, and resins: dissolved fraction) into higher-molecular-weight molecules (asphaltenes: precipitated fraction), thus increasing bitumen viscosity. Moreover, polar oxygen-containing groups form and tend to associate into micelles of higher micellar weight, increasing the viscosity of the bitumen (micelles: grouping of asphaltenes and heavier aromatic fractions of maltenes). Polar hydroxyl (-OH), carbonyl (C=O), and carboxylic (-C(=O)-OH) groups are formed during oxidation, resulting in larger and more complex molecules that make the bitumen harder and less flexible [33,38].

Quantifying bitumen oxidation requires chemical or physico-chemical tests to analyze structural, compositional, and molecular modifications caused by oxygen, considering that bitumen typically consists of 80–88% carbon, 8–15% hydrogen, 0–3% oxygen, 0–7% sulphur, and 0–1% other elements [33,39,40]. One commonly adopted tool to monitor the changes in the chemical composition of bitumen after aging and quantifying the oxidation products is Fourier-transform infrared (FTIR) spectroscopy [41]. This technique is used to obtain infrared absorption or emission spectra, over a wide spectral range, for solids, liquids, or gases [42]. In addition, FTIR spectroscopy has been used by a number of researchers to track changes in specific compounds as unmodified bitumen ages [43,44,45,46]. Most of these studies have observed the evolution of carbonyl (C=O) and sulphoxide (S=O) compounds from FTIR spectra after the aging of unmodified bitumen. Other compounds (or indices) such as aliphaticity (CH_2_ and CH_3_), aromaticity (C=C), and long chain ((CH_2_)_n_) were also monitored [45,47]. FTIR tests and their carbonyl and/or sulphoxide indices confirm that the Kraft lignins studied by these researchers reduce bitumen aging [29,30,31,32,48,49] or, at worst, have little to no effect on aging. Finally, extensive FTIR and chemical analyses reveal that a high content of phenolic compounds in lignin (combined with a low molecular weight and aliphatic hydroxyl group content) may slow down (or hinder) bitumen oxidation due to the donation of hydrogen atoms during the reaction and/or the capture (or consumption) of free radicals generated by the reaction and/or the decomposition of lignin at high temperatures [7,15,25,27,30,35,48,50,51,52,53,54,55].

## 2. Experimental Program

### 2.1. Scope and Objectives

The main objective of this study is to analyze the potential anti-aging property and stiffening effect of a softwood Kraft lignin, in a low dosage (<10% mass of binder), on a standard, unmodified bitumen adapted for cold regions of Canada (Montreal area). The scope is limited to thermo-oxidative aging.

Moreover, this study is part of a larger project, whose objectives were to measure the effect of this Kraft lignin with low- to high-mass dosages (5 to 30% compared to bitumen), as a substitute for bituminous binder, on these elements [24,56,57,58,59,60]: (1) the performance grade (PG) of two unmodified bitumens adapted for cold regions; (2) the chemo-thermal characteristics of an unmodified bitumen, coupled with the effects of mixing conditions with a low/mechanical mixer and high shear mixer (MM and HSM); (3) the rheological and chemo-thermal properties of an unmodified bitumen, associated with the effect of the Sasobit^®^ additive; (4) the compactability of hot and warm mix asphalt (HMA and WMA), associated with the effect of the Sasobit^®^ additive; (5) the compactability, moisture sensitivity, rutting, and low-temperature crack resistance of HMA; and (6) field test sections of HMA with or without reclaimed asphalt pavement (RAP).

### 2.2. Experimental Plan

**Figure 1:**
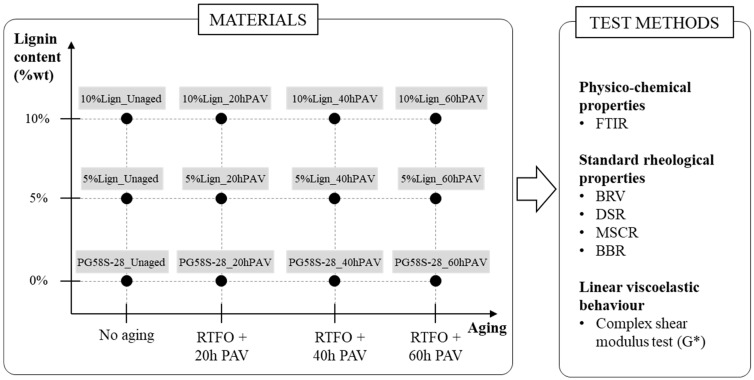
Scheme of the experimental plan.

The experimental plan of this study is laid out in Figure 1. From the same unmodified bitumen, a performance grade (PG) 58S–28, and the same Kraft lignin source, a total of twelve bitumens were produced to build a full 3 × 4 experimental matrix, with three (3) different lignin contents and four (4) different aging levels.

The three lignin contents of 0, 5, and 10% (y-axis: Figure 1), by the total mass of the bitumen blend, were chosen for two reasons. First, according to Zhang et al. [61] and Xu et al. [48], lignin can indeed show antioxidation properties at relatively low content (7–8%). Secondly, higher lignin content than 10% was not favoured in order to limit the stiffening from the “filler effect” of lignin on the bitumen. In the case of limited molecular solubility/compatibility, the lignin particles are expected to keep their shape and function as stiff inclusions in a softer matrix of bitumen, like any type of inert filler such as limestone, clay brick, or ceramic powders [62].

For each lignin content, the bitumens were subjected to four (4) levels of aging (x-axis: Figure 1): unaged and combined STA with RTFO and LTA with PAV for 20, 40, or 60 h. The bitumens were subjected to up to 60 h (a noticeably long period) of PAV in order to fully grasp the anti-aging potential of the lignin.

Each bitumen’s label “XXX_YYY” comprises two parts, separated by an underscore. The first part, “XXX”, indicates the composition, with three groups: “PG58S–28” for the unmodified bitumens and “5%Lign” and “10%Lign” for the bitumens modified with, respectively, 5 and 10% lignin. The second part of the label, “YYY”, indicates the aging level: “Unaged”, “20hPAV”, “40hPAV”, or “60hPAV”. Legends and references in the text can refer either to a group of bitumens (e.g., “5%Lign”) or a single specific bitumen (e.g., “5%Lign_Unaged”).

Details on the materials are provided in Section 2.3, whereas the sample preparation and sample aging procedures description are covered in Section 2.4 and Section 2.5. The same test methods, summarized on the right-hand side of Figure 1 and detailed in Section 2.6, were applied to all twelve bitumens to investigate the effect of lignin content and aging (including potential cross-effects) on three key bitumen aspects:

1. Physico-chemical properties, in our case the chemical composition by means of Fourier-transform infrared spectroscopy (FTIR), described in Section 2.6.1;
2. Standard rheological properties, obtained with the BRV, the DSR, the MSCR, and the BBR tests, described in Section 2.6.2;
3. The linear viscoelastic (LVE) behaviour, characterized by a complex shear modulus (G*) test, as described in Section 2.6.3.

### 2.3. Materials

A straight run, unmodified soft bitumen with a PG of 58S–28 was used for producing all tested bitumens. Its technical properties, provided by the manufacturer, are listed in Table 1. The tests to be carried out, the standards or test methods to be followed, and the requirements to be met for bitumen are all specified in standard 4101 issued by the Ministry of Transportation of Quebec (*Ministère des Transports et de la Mobilité Durable*: MTMD) [34]. An unmodified PG 58S–28 bitumen is typically recommended by MTMD and adapted in either base or surface course for the climatic zone related to the Montreal area (−28 to 58 °C) and for low- or standard (S)-traffic roads [63].

A softwood-derived Kraft lignin, produced from the black liquor of a Canadian pulp mill, is used in this study. Its properties, as provided by the supplier, are shown in Table 2. A density of 1.263 kg/m^3^ was obtained internally, determined in accordance with the ASTM C188 standard, which corresponds to the values given by the supplier. The density of lignin may be low, but it is higher than that of bitumen: 1.263 versus 1.026 kg/m^3^. This translates into respective lignin percentages of 4.1 and 8.3 by volume for starting mass percentages of 5 and 10 of lignin. Its moisture content also remained low in the laboratory (around 1–2%), determined in accordance with the standard CAN/BNQ 2501-170 of the *Bureau de normalisation du Québec* (BNQ) [65], which is comparable to the initial value given by the supplier. It can also be seen that the lignin is a fine powder, with most particles smaller than 149 µm. Additional in-house analysis, with a Mastersizer 3000^®^ instrument equipped with a Hydro EV dispersion unit, revealed instead that the particle size of the Kraft lignin powder fell within the range of 1 to 100 μm, with 80% of the particles between 5.47 μm (D10) and 48.7 μm (D90) [58]. Finally, Kraft lignin is made up of four (4) main components: carbon, oxygen, hydrogen, and sulphur. It will be interesting to see how lignin composition is perceived by FTIR tests (Section 3.1).

### 2.4. Sample Preparation Procedure

Sample preparation involves mixing the Kraft lignin with bitumen using an IKA^®^ RW16 basic overhead stirrer (Staufen im Breisgau, Germany), classified as a low-shear mixer. It should be noted that the bitumen used here, the PG 58S–28, is normally heated to 150 °C during hot mix asphalt production. Here, to compensate for the increase in viscosity generated by the addition of lignin, the bitumen is heated to around 165 °C in order to obtain a good coating of the lignin and a homogeneous mix [24]. Secondly, Kraft lignin was gradually poured and mixed in with a speed of about 900 ± 20 rotations per minute (rpm). This step takes a few minutes at most. Finally, after all the lignin was incorporated in the bitumen, the mixing was continued for 15 min. During the mixing, the temperature of the bitumen was maintained by a hot plate. At this mixing temperature, the bitumen, as a viscous fluid, coated the lignin particles without causing their decomposition, which was confirmed through microscopic observations conducted after mixing [24].

It should be noted that virgin bitumen, i.e., bitumen without added lignin, has not undergone this sample preparation. Considering the short duration of the latter, aging was expected to be negligible, especially in comparison to the designed aging procedure described below.

### 2.5. Sample Aging Procedure

The aging procedure for the samples comprises two stages. Firstly, just after preparation (Section 2.4), the bitumen mixtures are placed in glass tubes to be subjected to the RTFO test (equipment Despatch^®^ model RTFO, Lakeville, MN, USA), which is carried out at 163 °C for 85 min to simulate short-term aging (STA). Secondly, bitumen mixtures are placed in stainless steel pans and subjected to the PAV test (equipment ATS^®^ model PAV-V3, Butler, PA, USA) for 20, 40, or 60 h at a temperature of 100 °C and a pressure of 2.11 MPa to simulate long-term aging (LTA). Finally, the samples in stainless steel pans are heated to 150 ± 5 °C for 4 ± 1 min so that they can be poured into the beakers and moulds required for their characterization (Section 2.6).

### 2.6. Test Methods

#### 2.6.1. Characterization of Physico-Chemical Properties

The study of the physico-chemical properties of bitumen focused on the infrared absorbance, evaluated with a Fourier-transform infrared (FTIR) spectroscopy in attenuated total reflectance (ATR) mode. A Spectrum Two^TM^ FTIR Perkin Elmer^®^ was used (Waltham, MA, USA). Prior to each test, the ATR crystal was cleansed using limonene and isopropanol, and a background spectrum was captured. For tests, twenty (20) scans were recorded for each spectrum.

To avoid oxidation, the bitumen was scooped from its storage beaker with a warm metal spoon (around 60 °C) after scraping a 5 mm layer off the top surface. The sample was then pressed against the measurement system’s diamond crystal. The wavenumber spectrum ranged from 400 to 4000 cm^−1^, had an experimental 4 cm^−1^ resolution, and was mathematically interpolated to 1 cm^−1^ to perform smoother under-peak-area calculations. As bitumen is composed of many different molecules that are virtually impossible to quantify separately, indices based on functional groups or bonds were selected to evaluate and compare semi-quantitatively the chemical composition of different materials. Each functional group absorbs the IR radiation by vibrating at a specific mode and frequency. The resonance wavelength is slightly shifted because of the molecule’s branches linked to the elements of the functional group. The variety of molecules in bitumen results in broad, single, or more complex peaks in the absorbance spectrum. In this paper, five functional groups were selected, based on previous studies on aged bitumen by Nivitha et al. [41], which are aliphatics (CH_2_, CH_3_), carbonyls (C=O), sulphoxides (S=O), aromatics (C=C), and long chains ((CH_2_)_n_). The indices are determined by first measuring, on an absorbance versus wavelength graph, the area (*A_X-Y_*) between a particular bond’s peak and the background linearized baseline, between two predefined wavenumbers *X* and *Y*. The numerical integration method was a simple Riemann sum with a 1 cm^−1^ step. The indices were then calculated by normalizing each area by the sum of all areas measured (ΣA). The calculation for each index was carried out as follows [41]: aliphatics (calculated as the area between 1350 and 1510 cm^−1^ divided by the total peak area, ΣA), carbonyl (1678–1725 cm^−1^/ΣA), sulphoxide (1010–1043 cm^−1^/ΣA), aromatics (1535–1625 cm^−1^/ΣA), and long-chain, 715–733 cm^−1^/ΣA). The effects of lignin content and aging on the chemical composition were appraised by how much the carbonyl and sulphoxide indices, two well-known sensitive parameters, varied across bitumens.

#### 2.6.2. Characterization of Standard Rheological Properties

Four (4) standard rheological properties were evaluated for all bitumens:

- The viscosity (*η*) at very high temperatures was measured with a Brookfield rotational viscosity (BRV) test following the AASHTO T316 standard, carried out on an AMETEK^®^ DV2TLV viscometer (Berwyn, PA, USA) with the SC4-21 spindle. The test temperatures were 135, 150, and 165 °C.
- The stiffness at high temperatures was appraised with a dynamic shear rheometer (DSR) test following the AASHTO T315 standard, carried out on a Malvern DSR (model Kinexus) with a 25 mm diameter and 1 mm gap parallel plate geometry (Malvern, UK). The stiffness parameter |G*|/sinφ at 1.59 Hz was recorded at different temperatures, starting from 58 °C and increasing by 6 °C each step. For each bitumen, the last test temperature was set such as |G*|/sinφ, ending up lower than 1.00 kPa (up to 100 °C for the stiffest bitumen in this study), and the second to last temperature corresponds to the PG high temperature (*T_high_*). The stiffness parameter is derived from the norm of the complex shear modulus labelled “|G*|” and from the phase angle labelled *φ*. The reader should notice that other labels might be used for those properties in other sources, especially “G*” for the norm (often called “dynamic modulus”) and “*δ*” for the phase angle. Those were not selected to avoid confusion with the complex modulus analysis (Section 2.6.3). Following the AASTHO M 320 standard, the results of DSR tests were interpolated to obtain, respectively, the “critical”, or “continuous” PG high temperature (Equation (1)).

(1) $$T_{c,high}=T_{high}+6\left(\frac{\log\left(1.00\right)-\log{\left(G^{*}/sin\delta \right)}_{T_{high}}}{\log{\left(G^{*}/sin\delta \right)}_{T_{high}+6}-\log{\left(G^{*}/sin\delta \right)}_{T_{high}}}\right)$$

- The creep and recovery potential at high temperatures was evaluated with a multiple stress creep recovery (MSCR) test following the AASHTO T350 standard, carried out on the same test set-up as the DSR test. The non-recoverable creep compliance (*J*_*nr*,3.2_) at 3.2 kPa was recorded at least at 58, 64, and 70 °C. For stiffer bitumens, the test temperature range was extended with a 6 °C step increase to T_high_, when the latter was higher than 70 °C.
- Finally, the creep compliance at low temperatures was investigated with a bending beam rheometer (BBR) test following the AASHTO T313 standard and carried out on a Cannon BBR rheometer, model “TE-BBR”. The secant modulus *S*(60) and the creep rate or m-value *m*(60) after 60s were measured either at −12 and −18 °C (for stiffer bitumen) or −18 and −24 °C (for softer bitumen). The lowest test temperature for which two rheological criteria are met, *S*(60) < 300 MPa and *m*(60) > 0.300, is first determined. The PG low temperature (*T_low_*) is then defined as 10 °C below this test temperature. Hence, a bitumen that passed at −12 and −18, but failed at −24 °C, possesses a *T_low_* of −28 °C. According to the AASHTO M 320 standard, the results of BBR tests are interpolated to obtain the critical low temperatures associated with each criterion (Equations (2) and (3)).

(2) $$T_{c,low,S(60)}=T_{low}-6\left(\frac{\log\left(300\right)-\log{\left(S(60)\right)}_{T_{low}+10}}{\log{\left(S(60)\right)}_{T_{low}+4}-\log{\left(S(60)\right)}_{T_{low}+10}}\right)$$

(3) $$T_{c,low,m(60)}=T_{low}+6\left(\frac{0.300-{m(60)}_{T_{low}+10}}{{m(60)}_{T_{low}+4}-{m(60)}_{T_{low}+10}}\right)$$

The critical PG low temperature ($T_{c,low}$) is simply the most conservative of the two temperatures (Equation (4)). The difference between those two constitutes the “Delta T critical” parameter (${∆T}_{c,low}$) and is commonly used to evaluate how binders perform to non-load-related cracking and also to track how they respond to aging. ${∆T}_{c,low}$ is calculated as the difference between the temperature at which S reaches 300 MPa and the temperature at which the m-value reaches 0.300, as expressed in Equation (5). As bitumen ages, it becomes stiffer and less capable of stress relaxation, meaning that the secant modulus increases and the m-value decreases, which affects the ${∆T}_{c,low}$ value. Bitumens that are highly sensitive to aging will show a larger negative ${∆T}_{c,low}$ value.

(4) $$T_{c,low}=max\left[T_{c,low,S(60)},\;T_{c,low,m(60)}\right]$$

(5) $${∆T}_{c,low}=T_{low}\left(S\right)-T_{low}(m)$$

#### 2.6.3. Characterization of the Linear Viscoelastic Behaviour

The linear viscoelastic (LVE) behaviour was characterized with a complex shear modulus (G*) test, performed on the same Malvern^®^ apparatus (model Kinexus, Malvern, UK) as used for the DSR tests (see Section 2.6.2). The main feature of this test is that three different parallel plate (PP) geometries were combined to obtain precise experimental measurements over a wide range of temperatures (−30 to 82 °C) and frequencies (0.01 to 10 Hz). The control shear strain amplitude *γ*_0_ was also adapted for each geometry:

- Between −30 and −8 °C, 4 mm diameter with 2 mm gap PP, *γ*_0_ = 0.05% or 500 µm/m;
- Between −8 and 34 °C, 8 mm diameter with 2 mm gap PP, *γ*_0_ = 1% or 10,000 µm/m;
- Between 34 and 82 °C, 25 mm diameter with 1 mm gap PP, *γ*_0_ = 5% or 50,000 µm/m.

A preliminary amplitude sweep test was performed to control the stability of the complex modulus, making sure the viscoelastic behaviour of the bitumen remained in the linear domain. Particular care was required at the lowest temperature with the 4 mm parallel plate geometry, as the sample would always break before showing nonlinearity effects on G* with increasing loading amplitude. The shear strain amplitude was noticeably limited to unusual levels for bitumen testing (500 µm/m), but the high stiffness of the bitumen in these conditions still yielded a good shear stress signal resolution.

Regarding G* analysis, the time–temperature superposition principle (TTSP) was first assessed. If the TTSP was validated, the shift coefficients (*a_T,exp_*) were determined and then modelled (*a_T,WLF_*) with a William–Landel–Ferry (WLF) law, expressed in Equation (6). The 2S2P1D (2 Springs, 2 Parabolic, and 1 Dashpot) rheological model was chosen and calibrated to simulate the complex modulus (G*_2*S2*P*1*D_). It is a 7-parameter model, formalized in Equations (7) and (8). Details on those parameters and their relation to intrinsic material properties can be found in the original article [66]. Both WLF and 2S2P1D model coefficients were calibrated for a reference temperature *T_ref_* arbitrarily taken at 22 °C.

(6) $$a_{T,WLF}\left(T,\;T_{ref}\right)=\frac{C_{1}\left(T-T_{ref}\right)}{C_{2}+T-T_{ref}}$$

(7) $$G_{2S2P1D}^{*}\left(i\omega \tau_{G}\right)=G_{00}+\frac{G_{0}-G_{00}}{1+{\delta \left(i\omega \tau_{G}\right)}^{-k}+{\left(i\omega \tau_{G}\right)}^{-h}+{\left(i\omega \beta \tau_{G}\right)}^{-1}}$$

(8) $$\tau_{G}=\tau_{G,Tref}*a_{T}\left(T,\;T_{ref}\right)$$

The calibration of WLF coefficients (*C*_1_ and *C*_2_) was executed by minimizing the mean normalized error *MNE*(*a_T_*) given in Equation (9) with MS Excel^®^ Solver. The calibration of 2S2P1D coefficients is more complicated as there are up to 7 parameters to optimize, not only the norm of the complex modulus but also its phase angle. The calibration was first manual and then optimized with the solver to minimize firstly the mean normalized error on the norm of complex modulus *MNE*(|G*|) (Equation (10)), secondly the mean absolute error on the phase angle *MAE*(*φ*) (Equation (11)), and finally *MNE*(|G*|) a second time.

(9) $$MNE\left(a_{T}\right)=\frac{2}{N}\sum^{N}\frac{\left|a_{T,exp}-a_{T,WLF}\right|}{a_{T,exp}+a_{T,WLF}}$$

(10) $$MNE\left(\left|G^{*}\right|\right)=\frac{2}{N}\sum^{N}\frac{\left|\left|G_{exp}^{*}\right|-\left|G_{2S2P1D}^{*}\right|\right|}{\left|G_{exp}^{*}\right|+\left|G_{2S2P1D}^{*}\right|}$$

(11) $$MAE\left(\varphi \right)=\frac{1}{N}\sum^{N}\left|\varphi_{exp}-\varphi_{2S2P1D}\right|$$

where *N* is the number of experimental test points.

## 3. Results

### 3.1. Fourier-Transform Infrared Spectroscopy

**Figure 2:**
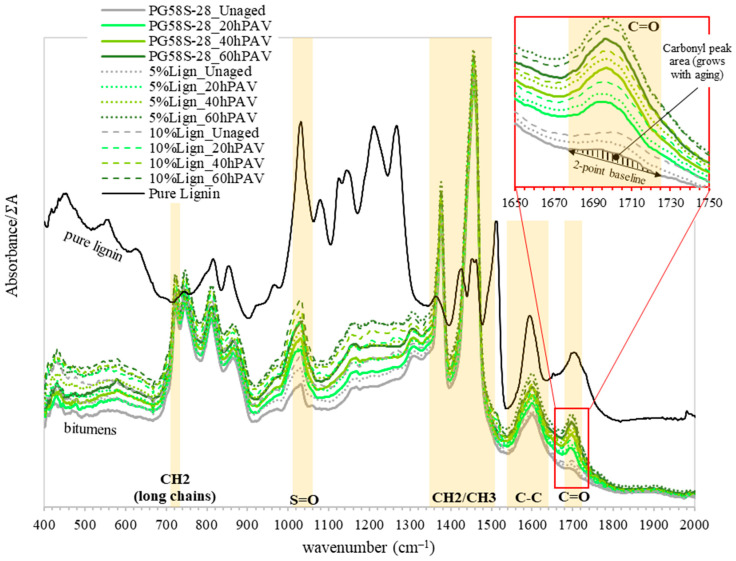
Normalized absorbance spectra of bitumens and pure Kraft lignin. Light yellow bands delineate the spectral bands for the area calculation between various peaks and their respective 2-point baselines.

The FTIR spectra of all bitumens and of the pure Kraft lignin are plotted in Figure 2. For better comparison, for each sample, the absorbance was normalized by the sum of peak areas Σ*A*, as defined in Section 2.6.1. The unit in the y-axis was removed since it is meaningless, and only the relative change in peak areas between material matters. A zoom on the carbonyl peaks is provided to illustrate the measurement of the peak area with a two-point baseline.

The biggest peak areas in bitumens are by far from the aliphatics (CH_2_/CH_3_) between 1365 and 1510 cm^−1^. They represent between 92 and 94% of the sum of peak areas (Σ*A*) for all twelve bitumens and appear to come primarily from the original PG58S–28 bitumen. This is convenient for the spectrum normalization process because this suggests that the lignin modification has little effect on the latter. In other words, the significant discrepancies observed in normalized peak areas across materials come from the actual chemical composition variation and not from a spectrum normalization bias.

From this overview of the spectra, it is clear that carbonyl (C=O) and sulphoxide (S=O) peaks, respectively, at the 1678–1725 cm^−1^ and 1010–1043 cm^−1^ bands, grow with aging. The carbonyl peaks are clearly identified and well delineated by the linear baseline. On the other hand, for the sulphoxide peak, the asymmetric shape suggests that several peaks overlap around 1010 cm^−1^. Therefore, the two-point baseline approach, as well as the current band selection, a simple spectral analysis, might limit the determination of a relevant index for the sulphoxides.

**Figure 3:**
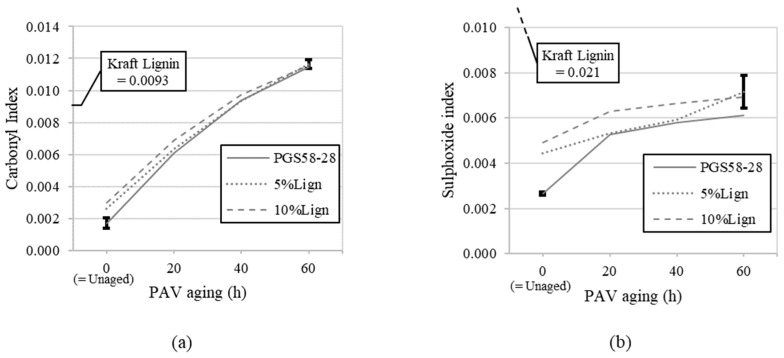
(**a**) Carbonyl indices and (**b**) sulphoxide indices as a function of PAV aging. Error bars represent standard deviations (SD) on the virgin PG58S–28 and on the modified and aged 10%Lign_60hPAV.

For a finer analysis, the carbonyl and sulphoxide indices are displayed as a function of aging in Figure 3.

The repeatability was tested on the virgin PG58S–28 and the aged, modified 10%Lign_60hPAV bitumens. For those two, error bars representing the standard deviation (SD) over three (3) FTIR sample repetitions were added to Figure 3. For the carbonyl index, the repeatability was excellent overall. The sulphoxide index showed more dispersion for the bitumen with 10% lignin and 60 h of PAV, making this index a less precise tool to evaluate the effects of lignin and aging.

A specific focus on the Kraft lignin reveals that the latter possesses a relatively high carbonyl index (0.0093) compared to the virgin PG58S–28 (0.0017), but it remains lower than that of all aged bitumens after 60 h of PAV (≈0.0116) (Figure 3a). The moderate increase in carbonyl observed for the unaged bitumens, namely from 0.0017 for the unmodified PG58S–28 to 0.0030 for the 10%Lign, can therefore be explained by the addition of lignin. As aging becomes more pronounced, so does its influence on the carbonyl index, reducing the relative discrepancy between unmodified and modified bitumens to a point where differentiation becomes impossible with this specific index. Hence, when tracking the evolution of the carbonyl index, nothing suggests that the bituminous phase of the lignin-modified bitumens undergoes less or more aging than in unmodified conditions.

The lignin exhibits a significantly higher sulphoxide index (0.021) compared to all tested bitumens (0.0027 to 0.0066) (Figure 3b). This creates a major discrepancy between the unmodified and lignin-modified bitumens, introducing a bias in the evaluation of the effect of aging or the assessment of lignin’s potential antioxidant properties. Although aging consistently increases the sulphoxide index, the limited magnitude of change and high variability reduce the reliability of this index for this study. The asymmetry of the peak aforementioned and presented in Figure 2 is also a source of bias. Extending the band from 1013–1043 to 985–1043 cm^−1^ to encompass the entire asymmetrical sulphoxide peak could help capture all the relevant absorbance contributions and improve the reliability of the sulphoxide index measurements. However, this does not mean that sulphoxide index analysis is irrelevant for other virgin bitumen or lignin sources.

Overall, the carbonyl index (Figure 3a) appears to be effective for tracking thermo-oxidative aging and there is no apparent anti-aging effect of lignin when considering the C=O bond intensity. The sulphoxide index (Figure 3b) seems less reliable due to higher variability in repeatability (SD) and the inherently high initial S=O bond content in pure Kraft lignin. It would be interesting to extend the chemical analysis of lignin-modified bitumens and assess the antioxidant properties of lignin with other aging mechanisms and/or conditions.

### 3.2. Standard Rheological Tests

The results of the standard rheological tests are summarized in Table 3.

The DSR results show that the incorporation of lignin, even at low dosages, induces a moderate stiffening effect at high temperatures (Table 3). For instance, in unaged conditions, $\left|G^{*}\right|/\sin\delta$ at 58 °C—10 Hz increases from 1.48 for the virgin PG58S–28 to 1.59 (+7.4%) and 1.69 kPa (+14.2%) for the modified bitumens containing 5 and 10% lignin, respectively.

**Figure 4:**
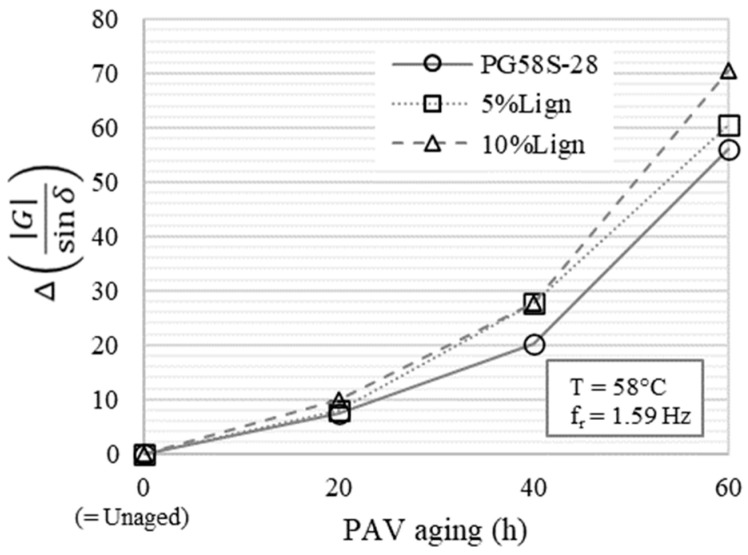
Relative change in $\frac{\left|G^{*}\right|}{\sin\delta }$ evaluated at 58 °C and 1.59 Hz as a function of PAV aging.

To better appraise the effect of aging, the relative change in the stiffness parameter $\left|G^{*}\right|/\sin\delta$ from the unaged condition is defined as (Equation (12)):

(12) $$∆\left(\frac{\left|G^{*}\right|}{\sin\delta }\right)=\frac{{\left(\frac{\left|G^{*}\right|}{\sin\delta }\right)}_{XhPAV}-{\left(\frac{\left|G^{*}\right|}{\sin\delta }\right)}_{Unaged}}{{\left(\frac{\left|G^{*}\right|}{\sin\delta }\right)}_{Unaged}}$$

where *X* represents the number of hours of PAV aging. This relative change in stiffness is evaluated at 58 °C—1.59 Hz, as plotted in Figure 4. Those test conditions provide a good stress and strain signal resolution across all bitumens despite having almost two orders of magnitude separating the stiffness of the unaged (min = 1.48 kPa) and of the most-aged bitumens (max = 120.8 kPa).

From those results, it is clear that Kraft lignin is not improving the aging resistance of the bitumens (Figure 4). If anything, the relative rigidification due to thermo-oxidative aging appears slightly stronger as the lignin content increases. This trend remains observable when higher test temperatures are considered.

**Figure 5:**
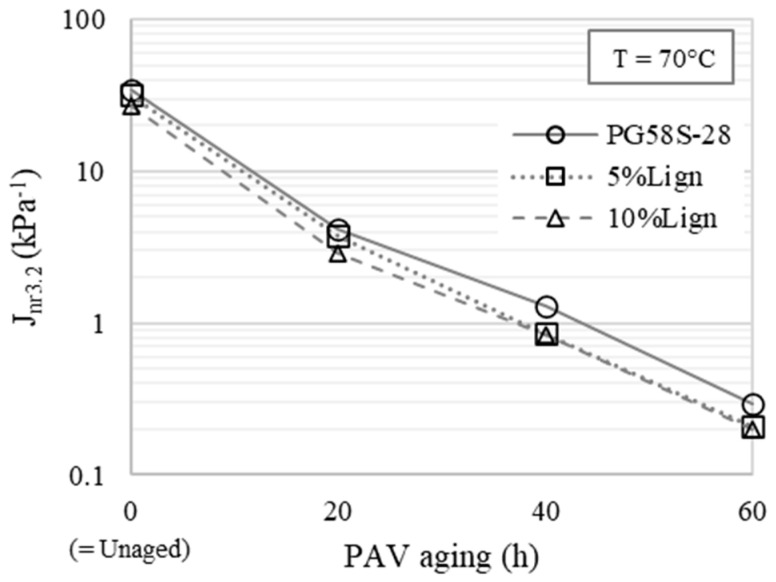
Non-recoverable creep *J*_*nr*,3.2_ evaluated at 70 °C as a function of PAV aging.

Similar observations are made with MSCR test results at high temperatures. The non-recoverable part of the creep compliance *J*_*nr*,3.2_ evaluated at 70 °C and displayed in Figure 5 allows the comparison between all bitumens with a good resolution. The stiffening effect of lignin modification alone is clearly visible, as the non-recoverable creep moderately decreases with respect to lignin content. The resistance to permanent deformation is primarily driven by thermo-oxidative aging. No anti-aging effect of lignin is apparent from the evolution of the resistance to permanent deformation, as the relative reduction in *J*_*nr*,3.2_ with aging is shown to be constant in the first approximation regardless of the lignin content. Indeed, non-recoverable creep curves are vertically translated on the semi-log graph in Figure 5. This trend remains observable when higher test temperatures are considered for material whose stress and strain signals are reliable (e.g., bitumens with 20, 40, and 60 h of PAV aging at 82 °C).

Regarding BBR test results at low temperatures (Table 3), the relative variations in secant modulus *S*(60) and creep rate *m*(60) at 60 s are defined subsequently as a function of aging (Equations (13) and (14)).

(13) $$∆S\left(60\right)=\frac{{S\left(60\right)}_{XhPAV}-{S\left(60\right)}_{Unaged}}{{S\left(60\right)}_{Unaged}}$$

(14) $$∆m\left(60\right)=\frac{{m\left(60\right)}_{XhPAV}-{m\left(60\right)}_{Unaged}}{{m\left(60\right)}_{Unaged}}$$

**Figure 6:**
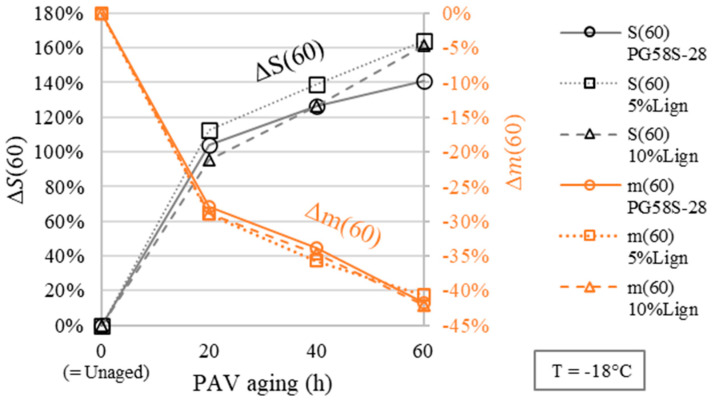
Relative change (Δ) of secant modulus *S*(60) and creep rate *m*(60) evaluated at −18 °C as a function of PAV aging.

Figure 6 presents those parameters evaluated at a test temperature of −18 °C for all bitumens. As expected, the thermo-oxidative aging induces a clear hardening of the bitumen (Δ*S*(60): black data in Figure 6). For instance, the secant moduli doubles after 20 h of PAV (96 to 113%). Meanwhile, no clear anti-aging effect of the lignin is observed. This is especially visible when tracking the relative variations—or even absolute variations—of the creep rate with aging (Δ*m*(60): orange data in Figure 6), the lignin-modified bitumens and the virgin PG58S–28 virtually share the same sensitivity to aging.

**Figure 7:**
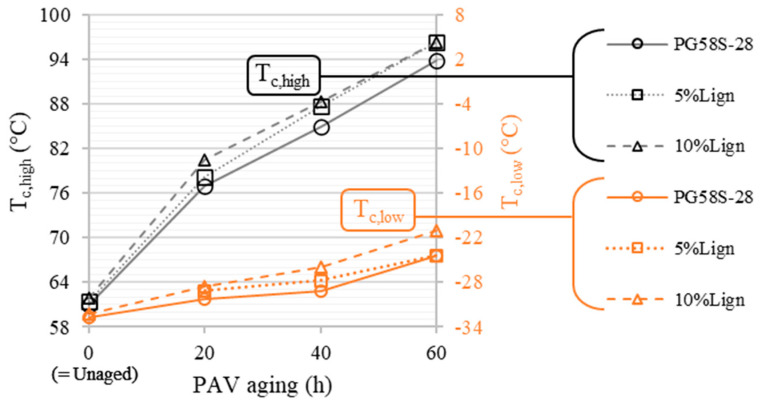
Critical high and low temperatures as a function of PAV aging (change in performance grade every 6 °C).

Both continuous PG temperatures are plotted as a function of aging in Figure 7. The thermo-oxidative long-term aging is the first-order parameter driving the increase in both *T_c,high_* and *T_c,low_*. The former jumps on average from 61.4 in unaged condition to 95.5 °C after RTFO + 60 h of PAV (gain of ≈5.5 grades at high temperature: *T_c,high_*), while the latter rises from −32.4 to −23.3 °C (loss of ≈1.5 grade at low temperature: *T_c,low_*). The results also show a second-order impact of lignin compared to aging, without any anti-aging cross-effect. If anything, it seems that adding lignin slightly amplifies the increase in high and low temperatures as a function of aging. For instance, the difference in *T_c,low_* between 10%Lign and PG58S–28 is only +0.5 °C in unaged conditions and ends up at +3.5 °C after 60 h of PAV. Meanwhile, the 5%Lign bitumen displays the same low temperature as the unmodified PG58S–28 after 60 h of PAV. It is difficult to establish any definite second-order trend on the anti-aging potential of Kraft lignin. Perhaps a specific study of the repeatability related to the aging and sample preparation processes, which were too heavy to carry out for this paper, could bring a more precise answer.

Additionally, the standard PG of a binder is obtained with *T_high_* in unaged conditions and *T_low_* after RTFO + 20 h PAV, both highlighted in grey in Table 3. The lignin modification of up to 10% of the total mass of bitumen was too limited to change the standard PG (58S–28).

In conclusion, the standard rheological tests show, in first approximation only, that Kraft lignin appears to act as any low-concentrated, inactive, or inert filler would: it provides a limited stiffening effect without providing any anti-aging property to the bitumen.

### 3.3. Complex Shear Modulus Test

**Figure 8:**
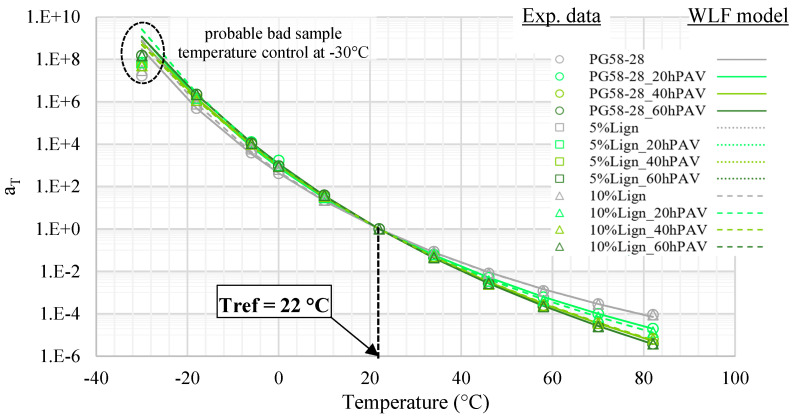
Experimental and WLF-modelled TTSP shift coefficients a_T_ as a function of temperature.

**Figure 9:**
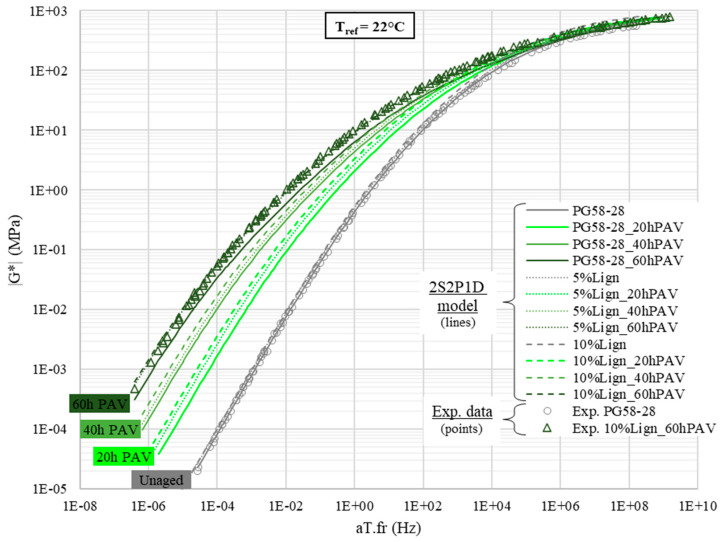
Master curves of the norm of complex shear modulus |G*|.

**Figure 10:**
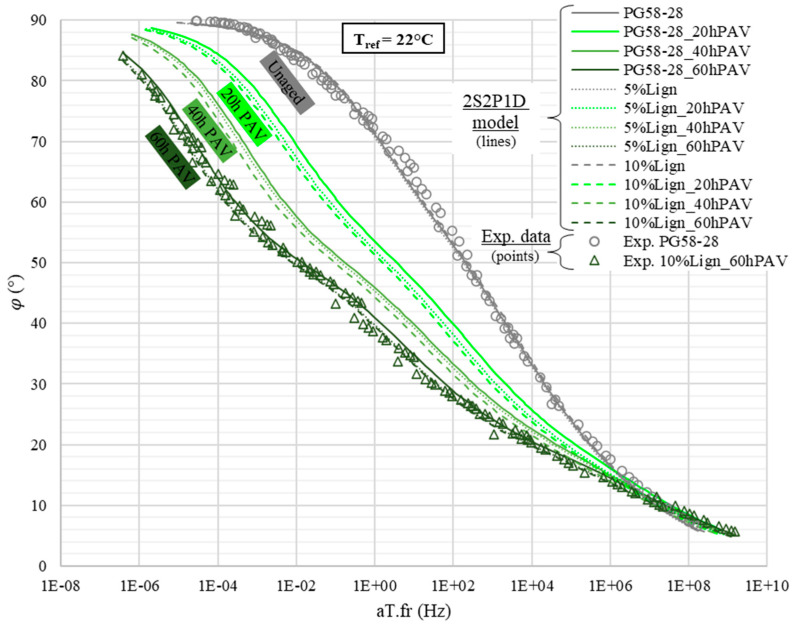
Master curves of the norm of phase angle *φ*.

The complex shear modulus was characterized in a broad temperature range, from −30 to 82 °C. Figure 8 presents the TTSP experimental shift coefficients and respective WLF models. The WLF parameters are listed in Table 4. The master curves of the norm and the phase angle of the complex modulus are, respectively, displayed in Figure 9 and Figure 10. For the sake of readability, only the experimental points of the virgin PG58S–28 and of the aged lignin-modified 10%Lign_60hPAV are plotted, leaving only the 2S2P1D models of the master curve for other bitumens.

The TTSP is verified for all bitumens, and the WLF model fits very well, with the sole exception of the lowest test temperature point of −30 °C (Figure 8). The most likely explanation is that the actual sample temperature was hotter than what the DSR probe indicated, due to limitations of the cooling system (note at top left). Indeed, as both the master curves of the norm and phase angles are continuous, the TTSP is still verified, and the experimental shift factor is therefore trustworthy. Were the actual material temperature closer to −25 rather than −30 °C, the experimental data points would match the model very well. Another possibility is the limitations of the WLF model itself near the glassy state transition, but this hypothesis would contradict classical results for bitumens [66].

Still at low temperatures, one of the experimental challenges was to obtain high-resolution strain and stress signals with the 4 mm PP geometry. After a lot of trial and error, the restriction to 500 µm/m on the shear strain amplitude (*γ*_0_) was necessary to avoid sample failure. Since all bitumens tend to converge to roughly the same glassy stiffness (1 GPa in shear mode: Figure 9), this load amplitude is recommended for similar studies using the 4 mm PP geometry. Additionally, the manual trimming has to be executed with particular care, since a sub-millimetric variation in the cross-section could have a non-negligible effect on the measurement of the sample stiffness [67]. Because of that lack of experimental precision at low temperatures, obtaining a precise estimate (e.g., more than one significant figure) of the glassy modulus *G*_0_ appears particularly difficult. Fortunately, thanks to the combination of the different DSR geometries, the complex modulus experimental data span over seven orders of magnitude and allow a thorough description of the LVE behaviour of all bitumens.

The constants of the model and the fitting errors are listed in Table 4. The 2S2P1D model fitted the experimental data very well. Indeed, the MNE(|G*|) was kept to a minimum, between 2.9 (PG58S–28_60hPAV) and 7.8% (10%Lign_60hPAV). Meanwhile, the *MAE(φ)* varied little between 0.9 (10%Lign_40hPAV) and 1.4° (PG58S–28_40hPAV). To put those results in perspective, a statistical study on the 2S2P1D calibration error by a 14-user panel on similar DSR test results (straight-run bitumen, tested from –30 to 60 °C and 0.01 to 10 Hz) found that the MNE(|G*|) can fall between 15 and 30%, and *MAE(φ)* between 1.3 and 3.5°, depending on the experience of the user (see, respectively, variables $\bar{∆{\left|G^{*}\right|}_{i}}$ and $\bar{∆\varphi_{i}}$ in Figure 8 of [68]). Thus, the 2S2P1D model was found to be perfectly fitted to model the low-dosage lignin-modified and/or strongly aged bitumens. The effects of lignin modification and aging on the LVE behaviour can be discussed not only via the visual comparison of the modelled master curves but also via a quantitative analysis of the variations in the 2S2P1D constants.

The comparison of the modelled master curves of the norm of the complex modulus (Figure 9) and of the phase angle (Figure 10) clearly reveals that adding 5 or 10% of lignin has a limited stiffening effect on the whole modulus spectrum. On the contrary, the aging induces a sharp increase in |G*| and a decrease in *φ* but not homogeneously across the spectrum. Indeed, at low and intermediate equivalent frequencies, the norm can change by more than an order of magnitude and the phase angle by 30° (Figure 10). For instance, at an equivalent frequency of 1.0 Hz (which corresponds to 22 °C—1 Hz), for unmodified bitumens, |G*| jumps from 0.4 to 10 MPa (Figure 9) and *φ* plummets from 72 to 41° (Figure 10) after RTFO + 60 h PAV. Meanwhile, at high equivalent frequencies, typically near the glassy state of all bitumens, the effect of aging becomes almost negligible, as complex moduli converge to purely elastic asymptotes (*G*_0_ = 1 GPa: Figure 9).

**Figure 11:**
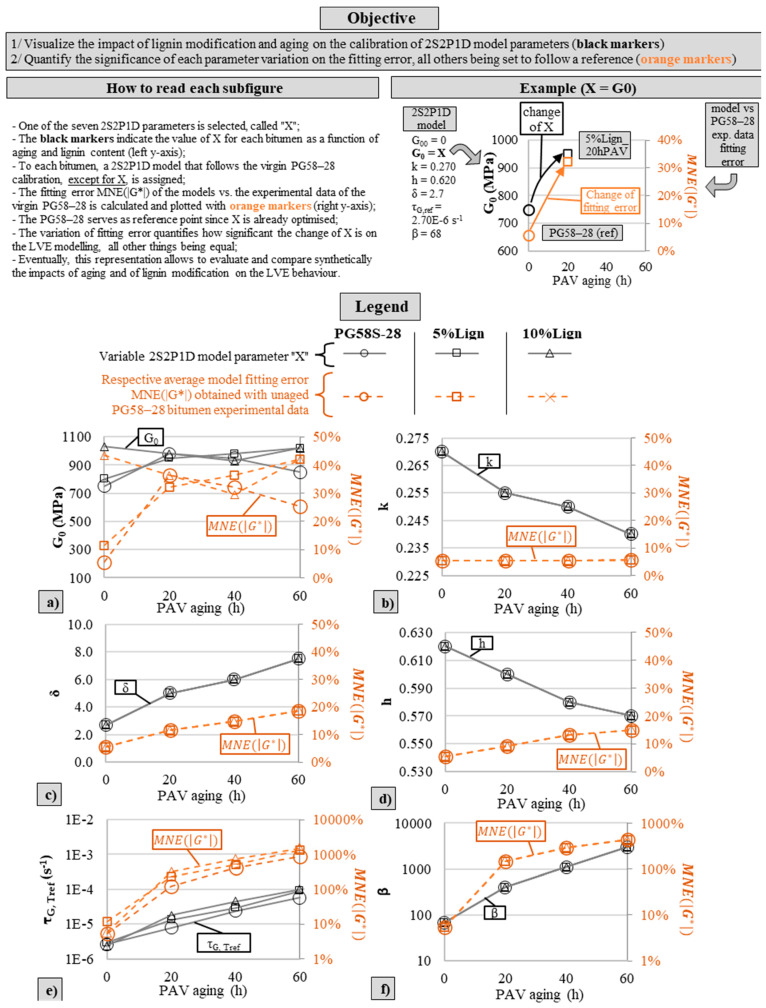
On the left-y-axis (black markers): 2S2P1D calibration parameters *G*_0_ in MPa (**a**), *k* (**b**), *δ* (**c**), *h* (**d**), *τ_G, Tref_* (**e**) in s^−1^, and *β* (**f**). On the right-y-axis (orange markers): MNE(|G*|) of the 2S2P1D model versus the unaged PG58S–28 experimental data, when only the associated left y-axis parameter varies while all other parameters follow the unaged PG58S–28 model calibration.

To elaborate on the impact of the lignin modification and aging, a quantitative analysis of each 2S2P1D constant is proposed in Figure 11. Each subfigure focuses on a given 2S2P1D parameter (listed in Table 4). Only *G*_00_ is excluded from the analysis since it is always null across all bitumens. For each subfigure, the associated 2S2P1D parameter is plotted as a function of aging and lignin content (dark markers, left y-axis in Figure 11). The mean normalized fitting error MNE(|G*|) of the 2S2P1D model versus the unaged PG58S–28 experimental data, when only the associated left y-axis parameter varies while all other parameters follow the unaged PG58S–28 model calibration, is also plotted (orange markers, right y-axis in Figure 11). In this configuration, the virgin PG58S–28 serves as a reference, since all its 2S2P1D parameters are already optimized to fit the experimental data, and therefore, the fitting error is minimized (MNE(|G*|) = 5.5%). For other bitumens, the variation in the fitting error from the reference represents the significance of the partial change in 2S2P1D calibration, parameter by parameter. Rather than discussing the absolute variations in an abstract parameter (e.g., *δ*), the reader can appreciate the relative significance of those variations on the global LVE behaviour model, which ultimately represents the real LVE behaviour with high fidelity, as discussed above.

With this new representation, it is possible to focus on how aging is affecting LVE behaviour. Although its contribution to the fitting error is important, the variation in the glassy modulus *G*_0_ (Figure 11a) is unclear, supposedly because of experimental precision limitations, as discussed previously. At first approximation, it seems that aging has no influence on that intrinsic material property. The change in *k* in Figure 11b, which is a key parameter at high equivalent frequencies [66], appears insignificant (the fitting error remains stable). The variations in *δ*, a shape parameter (Figure 11c), and *h*, a key parameter at low equivalent frequencies (Figure 11d), have a noticeable impact on the fitting error. The change in *β*, which drives the asymptotic viscous behaviour of the bitumen at the lowest equivalent frequencies (Figure 11f), is significant. Commenting on the characteristic time *τ_G_*, the only 2S2P1D parameter corresponding to the TTSP is complicated because the thermal susceptibility varies noticeably among tested bitumens. Not only the calibration parameter of interest is variable, namely the characteristic time at the reference temperature *τ_G, Tref_* (Figure 11e), but the TTSP shift factors as a function of temperature are different (Figure 8). Overall, aging is the primary factor in the change in LVE behaviour; the lower the equivalent frequency, the greater the effect.

A focus on the lignin modification clearly shows that the low-dosage lignin content has a very limited impact on the LVE behaviour and exhibits no anti-aging property when considering the whole G* spectrum. Indeed, four out of six 2S2P1D parameters, *k*, *h*, *δ* and *β* (respectively Figure 11b–d,f) are simply unaffected by the lignin content. Those constants are known to vary with the composition of the bitumen (with another crude oil source, with SBS polymer modification, etc.). *G*_0_ and *τ_G,Tref_* (Figure 11a,e) appear to increase only slightly without any clear cross-effect with aging. The variations in those two parameters are typical of the stiffening effect of inert fillers (e.g., limestone), as previous research on bituminous mastic has demonstrated [69]. This observation confirms and extends the previous conclusions on the limited stiffening effect and non-existing anti-aging potential of the Kraft lignin used in this study found with the standard rheological tests in Section 3.2.

## 4. Conclusions

The effects of long-term thermo-oxidative aging (up to 60 h of PAV) and of low-dosage Kraft lignin modification (up to 10% by total mass of bitumen) on the physico-chemical and rheological properties of a standard unmodified PG 58S–28 bitumen was studied. Several conclusions can be drawn:

- The FTIR carbonyl index appeared well-suited for tracking aging. However, no anti-aging effect of lignin could be detected with the proposed aging procedure. The sulphoxide index exhibited scattering due potentially to too many S=O bonds in the added lignin and to a limited spectral analysis method.
- The standard rheological tests showed that long-term aging primarily drove the rigidification of bitumen. Kraft lignin modification only provided a limited stiffening effect to the bitumen without any apparent anti-aging properties.
- The complex shear modulus (G*) tests successfully combined the 25, 8, and 4 mm parallel-plate geometries to obtain a precise, continuous spectrum. Experimental challenges at low temperatures with the 4 mm geometry were discussed. The most critical recommendation is the restriction of the shear strain amplitude (*γ*_0_) to 500 µm/m to avoid sample failure.
- For all bitumens, the time–temperature superposition principle (TTSP) was verified and the 2S2P1D model excellently fit the experimental data.
- A quantitative sensitivity analysis over the variations in the 2S2P1D constants showed that aging has a strong rigidification effect at low and intermediate equivalent frequencies, whereas it has virtually no impact at high equivalent frequencies, near the glassy state of bitumen. In parallel, the low-dosage lignin modification has a very limited stiffening impact on the LVE behaviour and exhibits no anti-aging properties.
- Eventually, the study concluded that the Kraft lignin used here at low dosages in this study could be assimilated to an inert filler in the first approximation. There is little to no apparent physico-chemical modification of the bitumen.

## 5. Future Developments

The authors propose several developments to further investigate the potential of Kraft lignin:

- Generalize the use and monitoring of LVE model parameters to characterize, quantify, and distinguish physical phenomena;
- Use a more in-depth chemical study (e.g., the influence of phenolic and other components) on the anti-aging potential of this lignin and other lignin sources and introduce other aging mechanisms such as exposure to light or ultraviolet rays, moisture, or various atmospheric gases;
- Study the anti-aging potential of lignin, an amorphous biopolymer, on polymer-modified bitumens;
- Use a compatibilizer for lignin to improve its interaction with bitumen (e.g., functional chains).

## Figures and Tables

**Table 1:** PG 58S–28 bitumen properties provided by the manufacturer.

Property	Method or Standard ^A,B^	Variable	Unit	Results
Density at 25 °C	ASTM D70	d_B_	g/cm^3^	1.0263
Rotational viscosity at 135 °C	AASHTO T316	η	mPa·s	254
Rotational viscosity at 165 °C	AASHTO T316	η	mPa·s	80
Storage stability	LC 25-003	-	°C	0.4
Ring and ball temperature	LC 25-003	T_RB_	°C	42.5
Flash point	ASTM D92	-	°C	305
Ash content	ASTM D8078	-	%	0.06
RTFOT mass variation	AASHTO T240	-	%	−0.415
Stiffness at 58 °C, unaged	AASHTO T315	|G*|/sin*φ*	kPa	1.24
PG high temperature	AASHTO M 320	T_high_	°C	59.7
PG low temperature	AASHTO M 320	T_low_	°C	−30.1
BBR secant modulus at −18 °C, unaged	AASHTO T313	S(60)	MPa	109
BBR creep rate, unaged	AASHTO T313	m(60)	-	0.496
Stiffness at 58 °C, RTFO aged	AASHTO T315	|G*|/sin*φ*	kPa	3.22
MSCR elastic recovery at 3.2 kPa and 58 °C, RTFO-aged	AASHTO T350	R_3.2_	%	0
MSCR non-recoverable creep at 3.2 kPa and 58 °C, RTFO-aged	AASHTO T350	J_nr,3.2_	kPa^−1^	3.05
MSCR non-recoverable creep difference at 58 °C, RTFO-aged	AASHTO T350	J_nr,diff_	%	9.6
BBR secant modulus at −18 °C, RTFO- + PAV-aged	AASHTO T313	S(60)	MPa	233
BBR creep rate, RTFOT + PAV aged	AASHTO T313	m(60)	-	0.344

^A^ AASHTO: American Association of State Highway and Transportation Officials; ASTM: American Society for Testing and Materials; ^B^ For further details on Québec test methods, please refer to the compendium of test methods (*Recueil des méthodes d’essai*) of the *Laboratoire des chaussées* (LC) of the MTMD [64].

**Table 2:** Properties of softwood Kraft lignin provided by the supplier.

Property ^A^	Variable	Unit	Results ^B^
Purity	-	%	95
Potential hydrogen	-	pH	3–4
Carboxylic acids content	-	mmol/g	0.53
Phenols content	-	mmol/g	2.62
Condensed phenols content	-	mmol/g	2.14
Aliphatic alcohols content	-	mmol/g	1.83
Density at 25 °C	d_L_	g/cm³	1.2–1.3
Moisture (water) content	%w	%	1
Passing of 100 mesh (149 μm)	-	%	99.3
Passing of 200 mesh (74 μm)	-	%	97.6
Glass transition temperature	T_g_	°C	162 ^C^
Decomposition temperature	T_d_	°C	150 ^D^
Ash content at 575 °C	-	%	0.42

^A^ Softwood Kraft produced from a Canadian pulp mill’s black liquor; lignin was obtained using the LignoForce^TM^ technology co-developed by FPInnovations (Pointe-Claire, QC, Canada) and NORAM (Vancouver, BC, Canada); the extraction process uses O_2_, CO_2_, H_2_SO_4_, and H_2_O; and lignin is made up of 64.6% carbon, 25.3% oxygen, 5.5% hydrogen, 1.5% sulphur, and 3.1% other elements. ^B^ Values provided by the supplier: no specific standards. ^C^ Determined by calculation. ^D^ Low-rate decomposition at this temperature and up to 200 °C.

**Table 3:** Standard rheological test results and performance grading of bitumens.

Test (Nbr. of Rep.)	Variable	Unit	T (°C)	PG58S–28				5%Lign				10%Lign			
				Un- aged	RTFO + 20 h ^A^	RTFO + 40 h ^A^	RTFO + 60 h ^A^	Un- aged	RTFO + 20 h ^A^	RTFO + 40 h ^A^	RTFO + 60 h ^A^	Un- aged	RTFO + 20 h ^A^	RTFO + 40 h ^A^	RTFO + 60 h ^A^
BRV (2)	η	(mPa·s)	135	342	784	1460	2203	375	913	1904	2737	421	1027	1772	2651
			150	170	352	629	900	179	412	797	1057	207	468	748	1032
			165	91	180	307	448	96	212	381	469	109	241	376	557
DSR (2)	|G*|/sinδ	(kPa)	58	1.48	12.46	31.46	84.62	1.59	14.48	45.81	97.97	1.69	18.70	48.36	120.80
			64	0.66	5.40	14.04	39.49	0.70	6.24	20.61	47.76	0.75	8.14	21.62	57.23
			70		2.41	6.31	18.44		2.79	9.26	22.90		3.64	9.72	26.17
			76		1.12	2.91	8.62		1.29	4.20	10.96		1.69	4.40	11.79
			82		0.56	1.40	4.06		0.64	1.98	5.30		0.83	2.08	5.39
			88			0.70	1.96			0.97	2.59			1.02	2.57
			94				0.98				1.29			0.53	1.28
			100								0.66				0.66
MSCR (2)	J_nr3.2_	(kPa^−1^)	58	8.074	0.580	0.151	0.027	7.552	0.556	0.090	0.021	6.390	0.394	0.085	0.019
			64	17.17	1.662	0.455	0.102	16.01	1.538	0.283	0.066	13.57	1.143	0.278	0.062
			70	34.37	4.191	1.286	0.292	31.59	3.758	0.849	0.208	26.88	2.916	0.835	0.198
			76		9.666	3.316	0.873		8.617	2.264	0.618		6.588	2.233	0.595
			82			7.609	2.347			5.294	1.668			5.114	1.630
			88				5.509							11.080	3.964
			94												8.690
BBR (3)	**S(60s)** *m(60s)*	**(MPa)** *(-)*	−12							**119**	**151**			**163**	**203**
										*0.347*	*0.317*			*0.329*	*0.293*
			−18	**110**	**225**	**250**	**266**	**121**	**258**	**290**	**320**	**144**	**281**	**325**	**376**
				*0.471*	*0.339*	*0.312*	*0.274*	*0.464*	*0.330*	*0.299*	*0.276*	*0.439*	*0.312*	*0.286*	*0.254*
			−24	**400**	**473**	**511**	**557**		**573**	**574**		**403**	**561**		
				*0.314*	*0.277*	*0.255*	*0.235*		*0.266*	*0.238*		*0.330*	*0.249*		
Perf. Grade	T_c,low, S(60)_	(°C)		−32.7	−30.3	−29.5	−29.0		−29.1	−28.3	−27.5	−32.3	−28.6	−27.3	−25.8
	T_c,low, m(60)_	(°C)		−34.7	−31.7	−29.1	−24.5		−30.6	−27.8	−24.4	−36.0	−29.0	−26.0	−21.0
	ΔT_c,low_	(°C)		2.0	1.3	−0.4	−4.5		1.5	−0.5	−3.1	3.7	0.5	−1.3	−4.8
	T_c,low_ ^B^	(°C)		−32.7	−30.3 ^B^	−29.1	−24.5		−29.1 ^B^	−27.8	−24.4	−32.3	−28.6 ^B^	−26.0	−21.0
	T_c,high_ ^B^	(°C)		60.9 ^B^	77.0	84.9	93.8	61.4 ^B^	78.1	87.7	96.3	61.9 ^B^	80.4	88.2	96.3
	**PG** ^B^	(°C)		**58S–28** ^B^				**58S–28** ^B^				**58S–28** ^B^			

^A^ Time in a PAV (Pressure Aging Vessel) at 100 °C and 2.11 MPa. ^B^ For obtaining the PG, T_high_ of unaged bitumen and T_low_ of RTFO + 20 h PAV are required (cells with grey shading).

**Table 4:** WLF and 2S2P1D constants.

**Model**	Variable	Unit	PG58S–28				5%Lign				10%Lign			
			Un- aged	RTFO	RTFO	RTFO	Un- aged	RTFO	RTFO	RTFO	Un- aged	RTFO	RTFO	RTFO
				+ 20 h ^A^	+ 40 h ^A^	+ 60 h ^A^		+ 20 h ^A^	+ 40 h ^A^	+ 60 h ^A^		+ 20 h ^A^	+ 40 h ^A^	+ 60 h ^A^
-	T_ref_	(°C)	22	22	22	22	22	22	22	22	22	22	22	22
WLF	C_1_		13.44	15.94	20.49	21.07	13.44	16.55	20.18	21.78	12.95	16.52	20.48	21.07
	C_2_	(°C)	134.30	143.27	172.68	172.88	134.30	143.22	172.77	179.17	127.67	143.16	174.55	172.91
2S2P1D	G_00_	(MPa)	0	0	0	0	0	0	0	0	0	0	0	0
	G_0_	(MPa)	750	980	950	850	800	950	980	1020	1030	980	930	1020
	k	-	0.270	0.255	0.250	0.240	0.270	0.255	0.250	0.240	0.270	0.255	0.250	0.240
	h	-	0.620	0.600	0.580	0.570	0.620	0.600	0.580	0.570	0.620	0.600	0.580	0.570
	δ	-	2.7	5.0	6.0	7.5	2.7	5.0	6.0	7.5	2.7	5.0	6.0	7.5
	τ_G,ref_	(s)	2.70 × 10^−6^	8.00 × 10^−6^	2.40 × 10^−5^	5.80 × 10^−5^	3.00 × 10^−6^	1.35 × 10^−5^	3.00 × 10^−5^	9.00 × 10^−5^	2.40 × 10^−6^	1.80 × 10^−5^	4.50 × 10^−5^	1.00 × 10^−4^
	β	-	68	400	1100	3000	68	400	1100	3000	68	400	1100	3000
	$MNE(|G^{*}|)$	-	5.5%	3.4%	4.4%	2.8%	5.6%	6.2%	2.9%	3.9%	6.2%	7.6%	7.8%	7.3%
	$\mathrm{Max}.\;|G^{*}|$ error ^B^	-	15.0%	12.5%	14.8%	9.4%	19.2%	13.3%	11.3%	12.3%	17.6%	17.2%	18.6%	29.9%
	MAE(φ)	(°)	1.0	1.0	1.4	1.1	1.1	1.1	1.2	1.1	1.0	1.1	0.9	1.2
	Max. φ error ^B^	(°)	3.8	4.1	5.1	3.0	3.5	3.9	5.2	3.6	3.3	3.7	6.3	4.5

^A^ Time in a PAV (Pressure Aging Vessel) at 100 °C and 2.11 MPa. ^B^ The highest fitting error with the 2S2P1D model found across all the experimental data points (absolute value).

## Data Availability

The original contributions presented in this study are included in the article. Further inquiries can be directed to the corresponding author.
